# A Case-control Study on Risk Factors for Severe Hand, Foot and Mouth Disease

**DOI:** 10.1038/srep40282

**Published:** 2017-01-13

**Authors:** Dingmei Zhang, Ruolin Li, Wangjian Zhang, Guowei Li, Zhanzhong Ma, Xiashi Chen, Zhicheng Du, Zhiyuan Li, Pi Guo, Zhuochen Lin, Jiahai Lu, Yuantao Hao

**Affiliations:** 1 School of Public Health, Sun Yat-sen University, Guangzhou, China; 2 Zhengzhou Center for Disease Control and Prevention, Zhengzhou, China; 3 Department of Clinical Laboratory, Yuebei People’s Hospital, Shaoguan, China

## Abstract

The objective of this study was to identify potential risk factors for severe hand, foot and mouth disease (HFMD). In this case-control study, 459 severe HFMD patients and 246 mild HFMD patients from Guangdong province and Henan province, China were included. Data comprising demographic characteristics, clinical symptoms and signs, laboratory findings and other factors were collected. Univariate analysis revealed 30 factors associated with severe cases. Further multivariate analysis indicated four independent risk factors: fatigue (*p* < 0.01, odd ratio [OR] = 204.7), the use of glucocorticoids (*p* = 0.03, OR = 10.44), the use of dehydrant drugs (*p* < 0.01, OR = 73.7) and maculopapular rash (*p* < 0.01, OR = 84.4); and one independent protective factor: herpes or ulcers in mouth (*p* = 0.01, OR = 0.02). However, more systematic research and validation are needed to understand the underlying risk factors for severe HFMD.

Hand, foot and mouth disease (HFMD) is a common infectious disease among young children. It is caused by genotypes of enterovirus species A, with the most frequently detected being enterovirus A71 (EV-A71), and Coxsackie A6, A10, A16^1^. In China, HFMD was first reported in 1981, Shanghai. After that, many provinces in China have reported HFMD cases^2^,^3^. HFMD can happen in any season and the peak time of incidence varies from area to area. In northern China the peak time is from April to June, while in southern China there is another peak that starts from September to November^4^. Enteroviruses spread through fecal-oral or oral-oral route: by eating foods or contact with toys contaminated by nasopharyngeal secretions or faeces from HFMD patients, or by inhaling droplets when HFMD patients are coughing or sneezing. Besides, some relevant studies also indicate that the prevalence of HFMD rises in pace with the rising temperature and relative humidity^5^. Similarly, the prevalent seasons of HFMD are spring and summer, when the weather is hot and wet and highly beneficial for enteroviruses to spread across the crowds^6^,^7^,^8^.

According to the national infectious disease direct network reports from China, from the year 2010 to 2012, the average annual incidence of HFMD was estimated 0.12%, about 1.1% of which developed into severe cases. Up to December 2015, the reported HFMD cases reached to 13.8 million^9^. The mortality of HFMD was 0.03%. In contrast, the mortality of severe HFMD was as high as 1.9%. Among those lethal cases, the detection rate of EV-A71 was 93%^10^. It is obvious that severe HFMD is of great harm to young children’s health and EV-A71 is considered to be the main pathogen of severe HFMD.

The occurrence of meningitis, encephalitis, cerebral spinal cord inflammation, acute flaccid paralysis, myocarditis, pulmonary edema and toxaemia is common among severe cases, especially nervous system involvement^11^,^12^. The average time for HFMD cases from onset to diagnosis, including the time for arriving at hospital and the time for diagnosis conformation, is about 1.5 days, while for lethal cases it is 3.5 days, and the average time from definite diagnosis to death is only 0.5 days^13^. At primary stage, usually there was no significant symptom for the severe HFMD cases, resulting in the delay of diagnosis and effective treatment. Therefore, early diagnosis is important during treatment of severe HFMD.

Moreover, there are no effective drugs to treat or prevent severe HFMD so far. Relevant studies indicated that more than 80% of the severe cases were among children under 6 years old. Other relevant risk factors include living in rural areas, long interval from the onset of illness to being hospitalized, serious conditions when patients are admitted to hospital, rashes occurrence, poor blood circulation, elevated blood glucose, elevated white blood cells, the infection of EV71, abnormal levels of the immune factors and cytokines and so on^13^,^14^. Simultaneously, the clinical manifestations of severe case including fatigue, startle, vomiting, irritability, abnormal white blood cell count, high blood glucose level, poor blood circulation of limbs, etc. recommended by Chinese National Ministry of Health for HFMD diagnosis and treatment guidelines 2008. However, these manifestations are mainly clinical test indexes, which can be the syndromes or outcomes of the severe cases and cannot be used to predict the outcome of HFMD cases at an early stage. Therefore, we conducted this retrospective case-control study to identify the potential risk factors for severe HFMD cases at an early stage.

## Results

### General characteristics of the participants

In total, 705 cases of HFMD were recruited, comprising 459 severe HFMD cases and 246 mild HFMD cases. The overall median age of the participants was 23.7 months (range 0–131 month) and the gender ratio was 1.9:1 (male: female). Table 1 outlines the demographic characteristics of all subjects including gender distribution, age distribution, the proportion of floating population and permanent resident population. Severe cases tended to be coming from the floating population and a significant difference was observed with respect to of position of residence. The general characteristics of cases and controls are summarized in Table 2. It shows that cases had significant higher birth weight, less breast-feeding, more allergic history and longer time interval between symptoms onset to being hospitalized. Besides, there were no statistical differences in the respect of other demographic and general characteristics among case and control groups.

### Univariate analysis

Clinical symptoms and signs, drugs use methods and laboratory findings of cases and controls are presented in Tables 3, 4 and 5. For clinical symptoms and signs, severe cases had a higher incidence of fever (≥37.3 °C), fatigue, limb trembling, dyspnea and vomiting, but a lower incidence of startle (move or jump suddenly in surprise or frighten). While the highest level of fever, twitch (sudden muscle spasm) and runny nose did not differ between severe cases and controls. Significant differences were also observed in the rash morphology and the position of their appearance. For severe cases, maculae and maculopapules were more frequently observed than herpes, but there was no difference with papule between case and control groups. Moreover, severe cases tend to have more rashes on the hips, ulcers on the cheeks, while having fewer ulcers in the mouth, rash on limbs, and ulcers on lips. Results from performed laboratory tests were analysed from both case and control groups (Table 4). Comparing with controls, cases tend to have higher white blood cell (WBC) count and neutrophilic granulocyte (NEUT) percentage but lower lymphocytes (LYM) percentage.

The therapeutic interventions given to participants from onset of HFMD until onset of severe complications were also obtained and summarized into five categories (Table 5). Cases and controls showed a significant difference with respects to all five therapeutic categories. Severe cases were more likely to use febrifuge (79.0% vs. 12.2%), glucocorticoid (92.4% vs. 15.9%), antivirotic (98.0% vs. 78.1%) and dehydrant drugs (87.2% vs. 2.9%), while non-severe cases had higher proportion using antibiotics (92.1% vs. 96.6%).

### Multivariate analysis

To determine the relative importance of the various factors associated with severe HFMD, we further performed a multivariate analysis. In the logistic regression model, maculopapular rashes, fatigue, the use of glucocorticoid and dehydrant drugs were significantly associated with increased risk of severe HFMD, with odd ratios (ORs) of 84.4, 204.7, 10.44, 73.7, respectively. On the other hand, herpes or ulcers in the mouth (OR = 0.02) was significantly associated with protections against severity (Table 6).

## Discussion

Although the majority of HFMD cases are generally mild and self-limiting, a small proportion of patients may rapidly develop severe complications which could be life-threatening^15^. Previous studies have shown that close monitoring and stage-based management program speculated with earlier admission of high risk groups, usage of IVIG, more advanced intensive care management may significantly decreased the case fatality rate^16^. Therefore, it is necessary to identify the risk factors, which may predict the occurrence of severe cases. This study aims to explore the risk factors that might indicate a child’s risk of progression to severe HFMD and to provide further information to help identify patients who may progress at an early stage.

In this study, risk factors for severe HFMD were fatigue, maculopapular rash, absent ulcers in the mouth and lower platelet count, the use of glucocorticoid and dehydrant drugs. It is also shown that demographic and general characteristics of patients did not affect the disease severity. Fatigue could be a marker for central nervous system involvement. According to the epidemic of EV-A71 in Taiwan in 1998, central nervous system involvement first developed in the most severe cases, followed by circulatory system involvement^17^,^18^. In the most severe cases, patients developed tachycardia and cyanosis within 2 to 5 days after onset of HFMD, and died within 12 to 18 hours after onset of these symptoms^19^,^20^. Therefore, children with fatigue should be early recognized and monitored closely in order to prevent fatal complications. Rash morphology and its position would also predict a more complicated or fatal HFMD. We found that the absence ulcers or herpes in the mouth and the presence of maculopapular rash were indicators of severe disease, which is consistent with previous studies^15^,^21^,^22^. However, in a recent meta-analysis^23^, different findings were presented, showing that there may be no association between ulcers in the mouth and the risk of progressing severe complications. Platelet count increase associating with severe HFMD was reported in some studies. In contrast, we found that comparing with severe cases, the platelet count of non-severe cases was significantly higher, while more of severe cases (53.82%) had normal platelet count.

We also found that the use of glucocorticoids and dehydrating drugs, which were used to ease up body’s response to inflammation and attenuate encephalic hypertension of the central nervous system of severe cases, was associated with an increased risk of severe HFMD. Our study results reinforce the previous findings that early use of glucocorticoids may increase the risk of subsequent severe or fatal HFMD^21^,^22^. It has been reported that early use of glucocorticoids was associated with critical complications of HFMD within the first 48 hours of onset^24^. Glucocorticoids may impair innate immunity by inhibiting the activity of the innate immune system and suppressing the secretion of diverse immune mediators^25^. Animal studies were also conducted, showing that mice given dexamethasone after exposure to EV-A71 develop substantially higher viral loads and virus was found in the brain^26^. Therefore, we suggest that administration of glucocorticoids for early treatment of HFMD may account for the development of critical complications. There is no further evidence that using dehydrating drug would increase the risk of developing severe HFMD.

Among twelve demographic and general characteristics, floating population, birth weight, breast-feeding, allergic history, and time interval between symptoms onset to being hospitalized were associated with severe involvement in the univariate analysis, but lost statistical significance in the final model. Besides, there were no statistical differences in the respect of other demographic and general characteristics among case and control group. Therefore, we assumed that population pattern may not influence the severity of the disease, which was consisted with some previous findings.

Besides, there are a few limitations in this study should be considered. Because of the limited sample size of the control group, the study did not use a matched method. In logistic regression model, 95% confidence intervals for each variable are large due to the deletion of miss values. There was no viral diagnostics included, which could not identify virus genotyping. Since the study is hospital-based, the sample source of our research is limited and may cause selection biases. As a retrospective case-control study, the recall bias may be a concern. However, to reduce recall bias, in this study, the data were mainly collected through medical record, while face-to-face interviews with children’s parents were adopted only when study-related information was missing in medical records.

In conclusion, our findings suggested that clinicians should be cautious if children are diagnosed with HFMD with maculopapules but without herpes or ulcers in the mouth and showing fatigue. Besides, the use of glucocorticoid in the early stage of the disease should be conservatively due to its potential harmful impact.

## Methods

### Study design and case definition

The study was designed as a retrospective non-matched case-control study. Clinical data from HFMD cases who were admitted between January 2010 and December 2014 were retrospectively collected from the Infectious Diseases Surveillance System of Henan Province and the Yuebei People Hospital in Guangdong in 2015. Face-to-face interviews with the patient’s parents or guardians were held during the patients’ hospitalization time. Children (under 14 years old) were diagnosed HFMD if they had at least one of the following features: maculopapular or vesicular rashes on the palms and/or soles and vesicles or ulcers in the mouth. A case of severe HFMD was defined as HFMD accompanied with the occurrence of at least one of the following complications: encephalitis, meningitis, acute flaccid paralysis (AFP), cardiorespiratory failure, or death. Encephalitis was characterized by the presence of an altered level of consciousness or hallucinations. Meningitis was defined as pleocytosis in cerebrospinal fluid analysis. AFP was characterized by the acute onset of a reflexive limb weakness. Cardiorespiratory failure was defined by the presence of respiratory distress, tachycardia, pulmonary edema, and pulmonary congestion on chest radiography. A control of mild HFMD was defined as a child diagnosed as HFMD, without any serious complications, selecting from the same population. Medical examination notification and informed written consent were obtained from all participants, and the study was approved by the Ethics Committee of the School of Public Health, Sun Yat-Sen University, Guangzhou City, Guangdong Province.

### Data collection

Data comprising demographic characteristics, general characteristics, clinical symptoms and signs, laboratory findings, and other factors that might be associated with or indicate severe and fatal HFMD were collected by trained interviewers through retrospective medical records in 2015 and face-to-face interviews with children’s parents using a standardized questionnaire during the patients’ hospitalization time. The clinical information of case subjects was collected from notes made after onset of illness, but before diagnosis of severity or fatality.

### Statistical analysis

Data were double-entered and validated using EpiData version 3.1 (the EpiData Association, Denmark) and analysed in R version 3.2.3 (R Development Core Team, 2015, R Foundation for Statistical Computing, Vienna, Austria). Differences in the means of continuous variables were tested using Student’s *t* test if the data were normally distributed and by Kruskal-Wallis otherwise. Pearson’s χ^2^ or Fisher’s exact test was used to test for differences in the proportions of categorical variables. For preliminary statistical analysis, 45 potential risk factors were tested for association with the severe HFMD using univariate logistic regression analysis. Variables showing a *p-*value < 0.05 were selected for inclusion in a multivariate logistic regression model built in backward stepwise fashion.

## Additional Information

**How to cite this article**: Zhang, D. *et al*. A Case-control Study on Risk Factors for Severe Hand, Foot and Mouth Disease. *Sci. Rep.* **7**, 40282; doi: 10.1038/srep40282 (2017).

**Publisher's note:** Springer Nature remains neutral with regard to jurisdictional claims in published maps and institutional affiliations.

## Figures and Tables

**Table 1:** The demographic characteristics of cases and controls.

Demographic characteristics^a^	Cases (n = 459)	Controls (n = 246)	*p*-value
Age, months(mean ± SD)	23.8 ± 14.8	23.6 ± 18.1	0.356
Male	299(65.1%)	162(65.9%)	0.976
Scatter children^b^	168(36.6%)	193(78.5%)	0.840
Floating population^c^	213(46.4%)	210(85.4%)	0.012
Position of residence			0.007
Rural area	172(37.5%)	134(54.5%)	—
Semi-urban area	13(2.8%)	11(4.5%)	—
Urban area	53(11.6%)	80(32.5%)	—

^a^Except where otherwise indicated, values are the number (percentage) of patients with the characteristic.

^b^Scatter children refer to children who are kept and raised at home instead of being put in day care.

^c^Floating population refers to migrants without local household registration status (hukou).

**Table 2:** Comparison of general characteristics between cases and controls.

General characteristics	Cases (n = 459)	Controls (n = 246)	OR (95% CI)	*p*-value
Premature birth				
Yes	19(4.3%)	10(4.2%)	1.0(0.5,2.3)	0.93
No	424(95.7%)	230(95.8%)	Reference	
Birth weight	3.5 ± 0.5	3.2 ± 0.4	2.9(2.0,4.2)	5*10^−9^(***)
Uterine-incision delivery				
Yes	253(56.9%)	150(62.8%)	0.8(0.6,1.1)	0.135(.)
No	192(43.2%)	89(37.2%)	Reference	
Breast-feeding				
Yes	312(70.3%)	221(92.5%)	0.2(0.1,0.3)	6*10^−10^(***)
No	132(29.7%)	18(7.5%)	Reference	
Complication during perinatal stage				
Yes	6(1.4%)	8(3.4%)	0.4(0.1,1.2)	0.099(·)
No	437(98.7%)	231(96.7%)	Reference	
congenital disease				
Yes	3(0.7%)	3(1.3%)	0.5(0.1,2.9)	0.44
No	444(99.3%)	236(98.7%)	Reference	
allergic history				
Yes	22(4.9%)	1(0.4%)	12.21(2.5,219,3)	0.015(**)
No	429(95.1%)	238(99.6%)	Reference	
Time interval between symptoms onset to being hospitalized (days)	3.6 ± 2.2	3.0 ± 1.7	1.2(1.1,1.3)	5.39*10^−4^(***)

Signif.codes: 0 ‘***’ 0.001 ‘**’ 0.01 ‘*’ 0.05 ‘.’ 0.1 ‘.’.

**Table 3:** Comparison of clinical symptoms and signs between cases and controls.

Symptoms and signs	Cases (n = 459)	Controls (n = 246)	OR (95% CI)	*p*-value
Fever (≥37.3 °C)				
Yes	455(98.7%)	217(91.2%)	7.3(3.1,20.1)	2.3*10^−5+^(***)
No	6(1.3%)	21(8.8%)	Reference	
Highest Level of fever (°C)	39.0 ± 0.6	38.9 ± 2.7	1.0(0.9,1.2)	0.69
Twitch				
Yes	32(7.0%)	9(3.7%)	2.0(1.0,4.5)	0.076
No	425(93.0%)	237(96.3%)	Reference	
Startle				
Yes	16(3.5%)	54(22.0%)	0.1(0.1,0.2)	5.8*10^−12^(***)
No	441(96.5%)	192(78.1%)	Reference	
Fatigue				
Yes	346(75.4%)	14(5.7%)	50.7(29.4,94.5)	3.1*10^−40^(***)
No	113(24.6%)	232(94.3%)	Reference	
Limb trembling				
Yes	288(62.9%)	13(5.3%)	30.4(17.5,57.4)	8*10^−30^(***)
No	170(37.1%)	233(94.7%)	Reference	
Runny nose				
Yes	24(5.2%)	17(6.9%)	0.7(0.4,1.4)	0.36
No	434(94.8%)	228(93.1%)	Reference	
Dyspnea				
Yes	61(13.3%)	4(1.6%)	9.3(3.8,30.9)	2*10^−5^(***)
No	397(86.7%)	242(98.4%)	Reference	
Vomiting				
Yes	190(41.4%)	29(11.8%)	5.3 (3.5,8.3)	3*10^−14^(***)
No	269(58.6%)	217(88.2%)	Reference	
Macula				
Yes	49(11.1%)	5(2.0%)	6.0(2.6,17.4)	0.0001(***)
No	394(88.9%)	241(98.0%)	Reference	
Papule				
Yes	37(8.3%)	30(12.2%)	0.7(0.4,1.1)	0.10(·)
No	407(91.7%)	216(87.8%)	Reference	
Maculopapule				
Yes	276(61.3%)	17(6.9%)	21.4(13.0,37.5)	6*10^−30^(***)
No	174(38.7%)	229(93.1%)	Reference	
Herpes				
Yes	215(48.3%)	183(76.3%)	0.3(0.2,0.4)	5*10^−12^(***)
No	230(51.7%)	57(23.8%)	Reference	
Rashes on hands				
Yes	412(90.2%)	217(88.2%)	1.2(0.7,2.0)	0.42
No	45(9.9%)	29(11.8%)	Reference	
Rashes on foot				
Yes	400(88.1%)	218(89.3%)	0.9(0.5,1.4)	0.62
No	54(11.9%)	26(10.7%)	Reference	
Rashes in the mouth				
Yes	144(32.1%)	173(72.7%)	0.2(0.1,0.3)	2*10^−22^(***)
No	304(67.9%)	65(27.3%)	Reference	
Rashes on hips				
Yes	291(64.4%)	119(48.4%)	1.9(1.4,2.7)	4.5*10^−5^(***)
No	161(35.6%)	127(51.6%)	Reference	
Rashes on trunks				
Yes	12(2.7%)	19(7.7%)	0.3(0.2,0.7)	0.003(*)
No	434(97.3%)	227(92.3%)	Reference	
Rashes on limbs				
Yes	19(4.2%)	32(13.0%)	0.3(0.2,0.5)	5.4*10^−5^(***)
No	429(95.8%)	214(87.0%)	Reference	
Ulcers or herpes in the mouth				
Yes	328(72.7%)	203(82.5%)	0.6(0.4,0.8)	0.003(*)
No	123(27.3%)	43(17.5%)	Reference	
Ulcers on cheek				
Yes	60(13.7%)	14(5.7%)	2.6(1.5,5.0)	0.001(**)
No	378(86.3%)	231(94.3%)	Reference	
Ulcers on buccopharyngeal				
Yes	125(28.3%)	59(24.1%)	1.2(0.9,1.8)	0.228
No	316(71.7%)	186(75.9%)	Reference	
Ulcers on lip mucosa				
Yes	45(10.3%)	70(28.6%)	0.3(0.2,0.4)	3*10^−9^(***)
No	394(85.8%)	175(71.4%)	Reference	

Signif.codes: 0 ‘***’ 0.001 ‘**’ 0.01 ‘*’ 0.05 ‘.’ 0.1 ‘.’.

**Table 4:** Comparison of laboratory findings between cases and controls.

Laboratory examinations	Cases (n = 459)	Controls (n = 246)	OR (95% CI)	*p*-value
WBC count(×10^9^/L)	10.6 ± 4.3	9.0 ± 2.8	1.1(1.1,1.2)	1.8*10^−5^(***)
NEUT(%)	54.5 ± 17.3	38.0 ± 17.8	1.1(1.0,1.1)	1.2*10^−19^(***)
LYM(%)	38.7 ± 16.3	428.6 ± 5022.6	1.0(1.0,1.0)	8.3*10^−16^(***)
RBC(×10^12^/L) count	4.4 ± 0.6	7.8 ± 36.9	0.77(0.54,0.96)	0.138
Platelet count	296.1 ± 116.8	369.8 ± 128.8	1.9(1.0,1.0)	5.7*10^−6^(***)

Signif.codes: 0 ‘***’ 0.001 ‘**’ 0.01 ‘*’ 0.05 ‘.’ 0.1 ‘.’.

**Table 5:** Comparison of therapeutic methods between cases and controls.

Drugs use methods	Cases (n = 459)	Controls (n = 246)	OR (95% CI)	*p*-value
Febrifuge				
Yes	362(79.0%)	30(12.2%)	27.02(17.6,42.8)	4*10^−48^(***)
No	96(21.0%)	215(87.8%)	Reference	
Glucocorticoid				
Yes	424(92.4%)	39(15.9%)	64.0(39.3,105.6)	3.4*10^−63^(***)
No	35(7.6%)	206(84.1%)	Reference	
Antibiotics				
Yes	422(92.1%)	230(96.6%)	0.41(0.2,0.9)	0.025(*)
No	36(7.9%)	8(3.4%)	Reference	
Antivirotic				
Yes	449(98.0%)	178(78.1%)	14.01(7.1,31.0)	1.4*10^−12^(***)
No	9(2.0%)	50(21.9%)	Reference	
Dehydrant drugs				
Yes	400(87.2%)	7(2.9%)	230.5(111.1,562,4)	1.4*10^−40^(***)
No	59(12.9%)	238(97.1%)	Reference	

Signif.codes: 0 ‘***’ 0.001 ‘**’ 0.01 ‘*’ 0.05 ‘.’ 0.1 ‘.’.

**Table 6:** Multivariate analysis on risk factors for severe hand-foot-mouth disease among children.

Risk factors	Odd Ratio (95% CI)	*p*-value
Maculopapule	84.4 (7.9, 2947.9)	<0.01(**)
Mouth ulcers	0.02 (0.00031, 0.29)	0.01(*)
Fatigue	204.7 (13.3, 16117.1)	<0.01(**)
Glucocorticoid	10.4 (1.2, 137.8)	0.03(*)
Dehydrant drugs	73.7 (6.2, 3327.4)	<0.01(**)
Platelet count	0.99 (0.98, 1.00)	<0.01(**)

Signif.codes: 0 ‘***’ 0.001 ‘**’ 0.01 ‘*’ 0.05 ‘.’ 0.1 ‘.’.
